# A Time Series Data Filling Method Based on LSTM—Taking the Stem Moisture as an Example

**DOI:** 10.3390/s20185045

**Published:** 2020-09-05

**Authors:** Wei Song, Chao Gao, Yue Zhao, Yandong Zhao

**Affiliations:** 1 School of Technology, Beijing Forestry University, Beijing 100083, China; Nintendo@bjfu.edu.cn (W.S.); zhaoyue0609@126.com (Y.Z.); 2 Beijing Laboratory of Urban and Rural Ecological Environment, Beijing Forestry University, Beijing 100083, China; 3 School of Computer and Information Engineering, Beijing Technology and Business University, Beijing 100048, China; gaochao9158@btbu.edu.cn

**Keywords:** data filling, LSTM neural network, missing data, stem moisture

## Abstract

In order to solve the problem of data loss in sensor data collection, this paper took the stem moisture data of plants as the object, and compared the filling value of missing data in the same data segment with different data filling methods to verify the validity and accuracy of the stem water filling data of the LSTM (Long Short-Term Memory) model. This paper compared the accuracy of missing stem water data for plants under different data filling methods to solve the problem of data loss in sensor data collection. Original stem moisture data was selected from *Lagerstroemia Indica* which was planted in the Haidian District of Beijing in June 2017. Part of the data which treated as missing data was manually deleted. Interpolation methods, time series statistical methods, the RNN (Recurrent Neural Network), and LSTM neural network were used to fill in the missing part and the filling results were compared with the original data. The result shows that the LSTM has more accurate performance than the RNN. The error values of the bidirectional LSTM model are the smallest among several models. The error values of the bidirectional LSTM are much lower than other methods. The MAPE (mean absolute percent error) of the bidirectional LSTM model is 1.813%. After increasing the length of the training data, the results further proved the effectiveness of the model. Further, in order to solve the problem of one-dimensional filling error accumulation, the LSTM model is used to conduct the multi-dimensional filling experiment with environmental data. After comparing the filling results of different environmental parameters, three environmental parameters of air humidity, photosynthetic active radiation, and soil temperature were selected as input. The results show that the multi-dimensional filling can greatly extend the sequence length while maintaining the accuracy, and make up for the defect that the one-dimensional filling accumulates errors with the increase of the sequence. The minimum MAPE of multidimensional filling is 1.499%. In conclusion, the data filling method based on LSTM neural network has a great advantage in filling the long-lost time series data which would provide a new idea for data filling.

## 1. Introduction

Water is the basis of plant metabolism and is an important component of plant cells [1]. The rapid acquisition of plants’ water status is of great significance for studies on plant physiological status and precision agricultural irrigation [2,3,4]. Plant water information is a kind of time series data, which is closely related to practical application and is also relevant to the field of data mining [5,6,7]. Plant information data is obtained through specific sensor monitoring [8]. However, due to equipment failure, power failure, severe weather, and other factors, the data loss rate is high [9,10]. Missing data is a great obstacle to the later use of data. Therefore, the filling of missing data is the premise and foundation of data mining, which has important application value [11].

Traditional data interpolation methods, such as the Lagrangian interpolation method [12], spline interpolation, piecewise cubic Hermite interpolation, mean value method, maximum frequency method, and principal component extraction method [13], can have high accuracy in filling a small number of scattered missing data. This paper explores solutions for the problem of long-term missing data caused by the failure of hardware equipment. The longer the missing data is, the lower the interpolation accuracy of the missing data is and the more difficult it is to fill [9].

In recent years, while maintaining the study of traditional methods, researchers have integrated elements of emerging fields and innovatively optimized the existing models. The filling method using a neural network [14] can be applied to any nonlinear relation function and has a self-learning ability. Still, it requires large-scale data samples, and overfitting and generalization problems have always existed. We can get the global optimal solution by using the support vector machine [15] to fill the data. This method requires less data scale compared to traditional neural networks. However, most methods based on support vector machines focus on single variable information, some of which involve manual judgment and rely on operators’ experience. Using the time series statistical model ARIMA to predict solar activity can predict the trend of change [16], but it is difficult to fit the fluctuation of the data. More and more people combine time series statistical models with neural network models, and use neural network models to process non-periodic components to improve accuracy. For example, the mixed model of ARIMA and ANN has obtained better prediction results [17,18]. The adaptive fuzzy neural network is used to predict the lane changing behavior of vehicles, and compared with the three traditional machine learning methods of neural network, support vector machine and multiple linear regression, the result is that AFNN has the best effect [19]. The hybrid model combining time series model and artificial neural network that predicts the wind speed in Brazil [20] is the most similar example to the situation in this paper: (1) The wind speed fluctuation shows regularity; (2) The predicted result in the experiment is more than one year and the span is long; (3) The prediction result has high precision, which can meet the needs of practical application. The above results prove the applicability of the neural network to time series data. For the case of this paper, a more appropriate neural network model can be selected for experiment. The ability of neural networks to deal with nonlinear problems is outstanding, and RNN has a strong advantage in analyzing time series problems [21]. Plant physiological state is gradual, which requires a neural network with a strong ability to remember historical information. The RNN model has been extensively studied in the field of language and text recognition [22]. The LSTM neural network model, optimized on the basis of RNN [23], has achieved good results in areas such as semantic analysis and image recognition that require strong historical information memory [24,25,26], but it is rarely used in the field of physiological data analysis. Time series data inevitably discuss cyclical and non-cyclical issues. The forecast for Freeway Speed focuses on the impact of periodicity on the forecast [27]. The prediction results of the six models in the article have been significantly improved after considering the cycle effects. And, considering the period component, the prediction step size and the use of a mixed model can significantly improve the prediction accuracy.

Based on the background that plant physiological information data is a dynamic time series data with obvious fluctuation, this paper selects the plant physiological information data filling method based on LSTM neural network. LSTM has the memory capacity to cope with long periods of data loss and performs better in the field of regularly fluctuating data. The LSTM model has a strong advantage in non-periodic analysis, and its memory capability also ensures high precision in periodic components. Data filling based on the LSTM model can reduce the influence of human factors and expand the length of the filling sequence. Using LSTM to fill data in two different environments, one-dimensional and multi-dimensional, has achieved better results than the traditional data filling method.

## 2. Materials and Methods

### 2.1. Introduction to LSTM Model and RNN Model

The LSTM (Long Short-Term Memory) model was proposed by Hochreiter et al. at the end of the 20th century [28]. LSTM is created based on RNN (Recurrent Neural Network). RNN is a model developed to deal with sequence problems, in order to better deal with information related to different inputs. Therefore, RNN was first used in the field of natural language processing.

**Figure 1:**
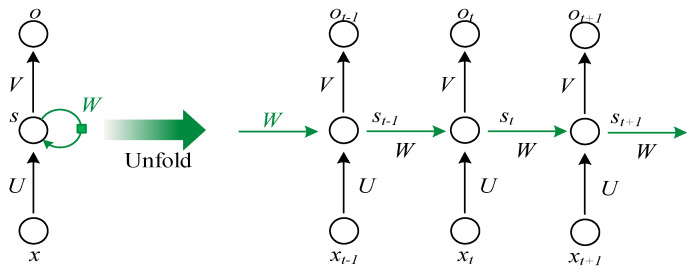
Unfold of RNN (Recurrent Neural Network).

Compared with the most basic fully connected neural network, RNN has a hidden layer of last state as input. That is, the value s of the hidden layer of RNN depends not only on the current input x, but also on the value s of the last hidden layer. Therefore, RNN has the ability remember due to variables from previously hidden layers. This unique network structure is shown in Figure 1.

RNN has memory ability, but due to the disappearance of the gradient and gradient explosion in long-term memory [29], the influence of the t-3 moment is very small on the state of t moment, which means that the network ignores the state before t-3 moment in training.

**Figure 2:**
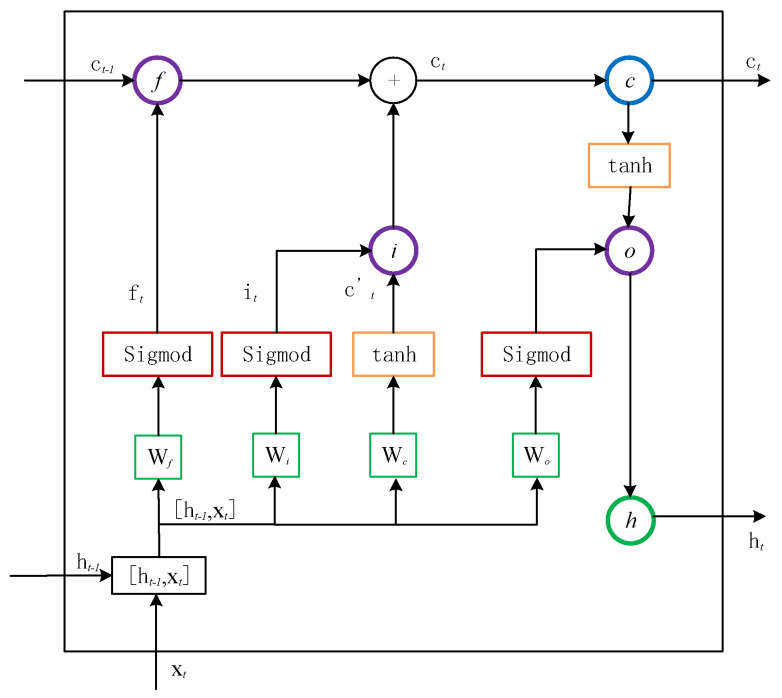
LSTM (Long Short-Term Memory) unit: f is the forgetting gate, i is the input gate, o is the output gate, c is the cell and h is the hidden layer state, b is the bias term, W is the weight matrix.

The LSTM long and short time memory network adds the unit state on the basis of RNN, that is, at a certain moment, the input of LSTM has three inputs: current time network input value x_t, the output of LSTM at the previous time h_(t-1), and unit state at last time c_(t-1). LSTM has two output values: current LSTM output value h_t, unit state at the current moment c_t. Compared with the traditional RNN, the neural unit of LSTM is a unit structure with a unique memory pattern. The cell structure of LSTM is shown in Figure 2. The memory unit in LSTM has three gates: INPUT GATE, FORGET GATE, and OUTPUT GATE. The content of the oblivion gate and input gate control unit state c_t: The forgetting gate determines the retention degree of the current state c_t to the cell state c_(t-1) at the previous moment, The input gate determines the retention degree of the current state c_t to the input x_t, and The output gate controls the degree to which c_t outputs to h_t in the current state.

(1) $$f_{t}=\sigma \left(W_{f}\cdot \left[h_{t-1},x_{t}\right]+b_{f}\right),$$

(2) $$f_{t}=\sigma \left(W_{f}\cdot \left[h_{t-1},x_{t}\right]+b_{f}\right),$$

(3) $$c_{t}=f_{t}\circ c_{t-1}+i_{t}\circ tanh(W_{c}\cdot \left[h_{t-1},x_{t}\right]+b_{c}),$$

(4) $$o_{t}=\sigma \left(W_{o}\cdot \left[h_{t-1},x_{t}\right]+b_{0}\right),$$

(5) $$h_{t}=o_{t}\circ tanh(c_{t}),$$

where *σ* is the activation function of Sigmoid, *f* is the forgetting gate, *i* is the input gate, *o* is the output gate, *c* is the cell, *h* is the hidden layer state, b is the bias term, *W* is the weight matrix.

The final output of LSTM is determined by the value of the output gate and the unit state.

### 2.2. LSTM Model Construction

There are three layers in the selected neural network and the number of neurons in each layer is 2 to the exponential power, which is 256, 128, and 64, respectively, as shown in Table 1.

#### 2.2.1. Calculation of Parameters in the First LSTM Layer

The input sample of network training is the matrix of (200, 1), that is, the time step is 200, and the characteristic length of each time step is 1. After each time step passes through the first LSTM layer, the output characteristic length is changed to 256 (i.e., 1→256), and the data of (1, 256) is obtained. Each LSTM neuron is divided into the forgetting gate layer, status update layer, and output gate layer. The following section calculates the specific parameters in each layer according to different layers.

Forgetting gate layer: in Formula (1), $h_{t-1}$ is the implicit vector of the previous state, namely, the implicit vector with a characteristic length of 256; $x_{t}$ is the input of the current state, namely the vector with characteristic length of 1, so the length of $\left[h_{t-1},x_{t}\right]$ is 256 + 1 = 257. The output length of this layer is 256. The activation function σ does not affect the dimension of the matrix.$\;\left[h_{t-1},x_{t}\right]\;$ is the matrix of (1, 257), and the matrix of (1, 256) is obtained by multiplying it with $W_{f}$, that is, the matrix of (257, 256) is $W_{f}$. After multiplying the matrix of (1, 256), add it to the bias matrix $\;b_{f}$, so $b_{f}$ should also be the matrix of (1, 256). The number of parameters of this layer is:

$$Param_{1}=\left(256+1\right)\times 256+256=66,048$$

Status update layer: Formula (2), Formula (3), and Formula (1) are similar. σ and tanh are activation functions and do not affect the number of parameters. The four values in Formula (3) are all known values, so there are no parameters to participate in the calculation, so the number of parameters in the status update layer is twice the number of parameters in the oblivion layer:

$$Param_{2}=66,048\times 2=132,096$$

Output gate layer: Formula (4) is the same as Formula (2), and the number of parameters involved in calculation is 66,048; Formula (5) and Formula (3) are the same, and those involved in the calculation are known values. Therefore, the number of parameters involved in the calculation of the output layer is the same as that of the forgetting gate layer:

$$Param_{3}=Param_{1}=66,048$$

In conclusion, the number of LSTM layer parameters in the first layer can be obtained:

$$Param_{LSTM1}=Param_{1}+Param_{2}+Param_{3}=264,192$$

It is observed that the number of LSTM layer parameters per layer is 4 times the number of forgetting gate layer parameters:

Param=4×((n+m)×m+m)

where *n* is the input of the current state and *m* is the output of the previous state.

#### 2.2.2. Calculation of Parameters in the Second LSTM Layer

According to the formula summarized above, the number of parameters in the second layer LSTM layer can be derived:

$$Param_{LSTM2}=4\times \left(\left(256+128\right)\times 128+128\right)=197,120$$

#### 2.2.3. Calculation of Parameters in the Third LSTM Layer

According to the formula summarized above, the number of parameters in the third layer LSTM layer can be deduced as follows:

$$Param_{LSTM3}=4\times \left(\left(128+64\right)\times 64+64\right)=49,408$$

The last layer is the full connection layer, with 64 neuron connections plus 1 bias:

$$Param_{dense1}=64+1=65$$

#### 2.2.4. Dropout Layer

The dropout layer in Table 1 is a network layer after the first LSTM layer to solve the over-fitting problem [30]. In neural network training, the training time and overfitting are the key problems that must be optimized and solved. Taking too long will reduce the efficiency of the model. Even if the accuracy is high enough, low efficiency can prevent the model from being practically useful. The dropout layer can solve both problems.

**Figure 3:**
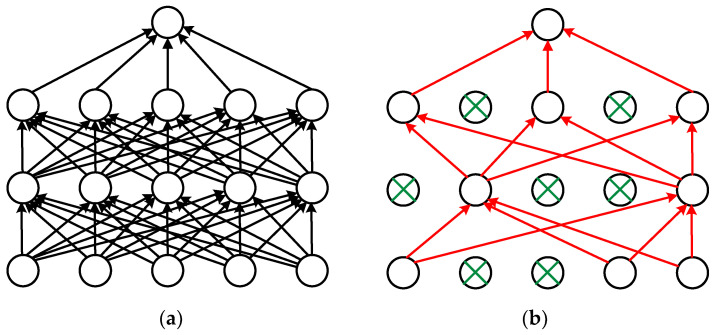
(**a**) is the standard network, and (**b**) is applying dropout. This time the green neurons were not involved in the training.

The dropout layer effect as shown in Figure 3: During model training, a certain proportion of nodes in the hidden layer are randomly excluded from the calculation. Although these nodes did not participate in the calculation this time, their weight is saved. The next cycle will be random again, and the nodes participating in the calculation will participate in the calculation with the weight obtained last time. Due to the small number of participating nodes, the calculation amount is greatly reduced, and the training time of the model is shortened. The nodes selected randomly in each cycle are different to avoid the overfitting caused by the unified calculation of all nodes. Since different nodes cannot be guaranteed to appear at the same time, it can avoid the situation that the network shows specific characteristics for a specific input, and it is more general.

### 2.3. RNN Comparison Model Construction

An RNN model with the same size as the LSTM model was established for a comparative experiment. The overview of RNN model is shown in Table 2. It can be seen from the overview of the model that, even with the same number of neurons, the number of parameters in the RNN model is much less than that in the LSTM model due to the difference in the structure of neurons, which is about 1/4 of the number of parameters in the LSTM model with the same structure.

The parameter calculation formula of LSTM model is as follows:

Param=4×((n+m)×m+m)

However, there is only one state in the hidden layer of RNN neurons, that is, there is no need to multiply by 4 in the outermost layer:

Param=(n+m)×m+m

where n is the input of the current state and m is the output of the previous state.

Therefore, the number of parameters in each layer of RNN is 1/4 of the number of parameters in each layer of LSTM, the total connection layer is 64, and the total number of parameters is about 1/4. The difference in the number of parameters is reflected in both training time and filling accuracy. Input 1000 data for 100 iteration training, and the training time of the LSTM network is 5–6 times that of RNN network.

### 2.4. The Data Source

The data used in this paper were selected from the stem moisture data of normal growing *Lagerstroemia Indica* trees 80 cm above the ground, collected in June, and planted in Haidian district of Beijing. Air temperature and humidity were measured using a HMP50-L6 sensor from Finland Vaisala (temperature: −10 to 50 °C, ±0.25 °C; humidity: 0–100%, ±2%). Photosynthetically active radiation was measured using a LI-190R sensor (0–10,000 µmol/(m^2^·s), ±5%) from Li-Cor, USA. The stem moisture content was measured using the BD-IV plant stem moisture sensor (0–60%, ±1%) of Beijing Forestry University. Data collection frequency is every 10 minutes intermittent collection, and 144 data per day. The data source information is shown in Table 3.

## 3. Results and Discussion

### 3.1. Result Analysis of One-Dimensional LSTM Model

#### 3.1.1. Model Training Result Analysis

**Figure 4:**
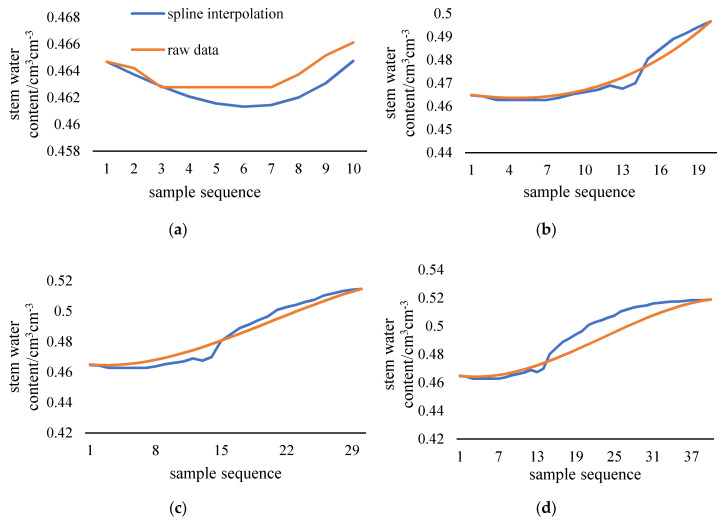

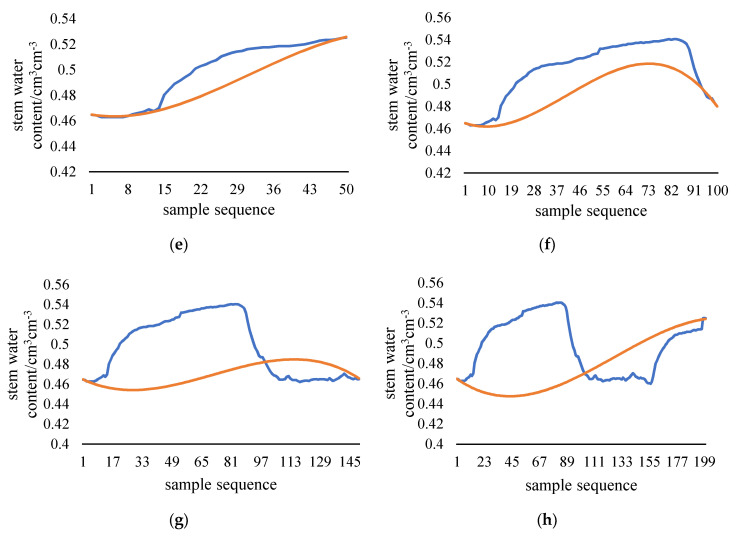
Interpolation filling results of different lengths. The length of filling is 10 (**a**), 20 (**b**), 30 (**c**), 40 (**d**), 50 (**e**), 100 (**f**), 150 (**g**), 200 (**h**), in this order.

As shown in Figure 4, spline interpolation method can’t fit the plant stem moisture when the data length exceeds 100. The longer the filling sequence is, the worse the fitting will be. Since there is no obvious fluctuation of stem moisture when the sequence length is less than 100, interpolation filling method can meet the requirement of data filling when the sequence length is short. This paper discusses the filling method of long-term series data that cannot be filled by interpolation method. Therefore, the length of the filling data should include at least a complete diurnal variation of plant stem moisture. Sensor data collection quantity is 144 data per day, 1008 data per week. The training data length is taken as 1000 adhering to the principle of taking integers as much as possible. The test data length is 200 which is larger than 144 and is an integer multiple of 100. The ratio of training data to test data is 5:1. The batch size of model is set to 100. It can be considered that one day of data is filled with one week of data.

**Figure 5:**
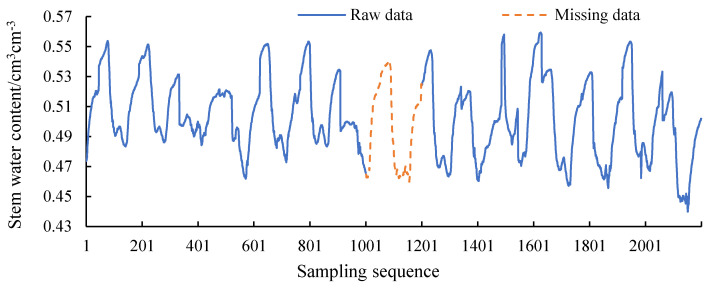
Series data of plant stem moisture.

In order to ensure the uniformity of data characteristics, the data selected in the article are all data from May to October. At this stage, the trees have entered a stable growth period, and the stem moisture presents regular fluctuations. The original data will be artificially divided into training and test sets. The training set is used for model training. The test set is used to compare with the model output to verify the performance of the model. A total of 2200 pieces of data are randomly taken from the database, as shown in Figure 5. The abscissa is the data order; the ordinate is the moisture content of the stem of the plant returned by the sensor, and the water content of the stem is from 43% to 57%. The data in the range of 1201 to 1200 is selected as the test data, the data length is 200, and the yellow dotted line is marked. The test data is in the middle of the 2200 pieces of data, ensuring that there are 1000 pieces of training data in both forward and backward filling. Suppose that the yellow dotted line represents a time period when there is a problem with the sensor or collector and other equipment, resulting in communication interruption and the data cannot be collected. The problem to be solved in this paper is to fill this missing data as accurately as possible. Through the data trend of Figure 5, it can be seen that the stem moisture of the plant is affected by the external environment, and fluctuates regularly with the sunrise and sunset cycles. The specific conditions of stem moisture at a certain moment will be affected by the specific external environment, but the overall fluctuation trend remains unchanged. Regularly varying data in time series is suitable for training and filling of LSTM networks, which is consistent with the original idea.

**Figure 6:**
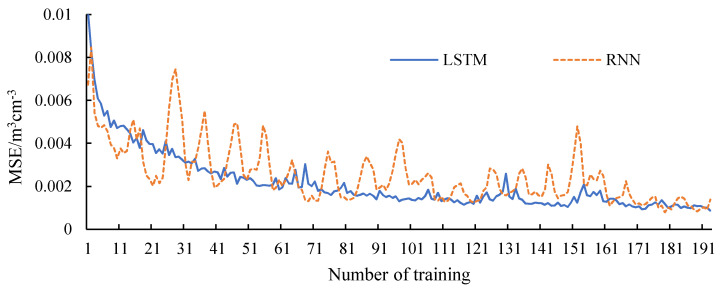
Model training error.

Figure 6 shows the change of loss during the training of LSTM and RNN. It can be seen that the loss value of the LSTM model decreases steadily throughout the whole process. The loss value of the RNN model has a general trend of decline, but it fluctuates sharply in the process of decline. The loss function is the mean-square error (MSE), indicating the deviation between the filling value and the real value during training. Since LSTM model has better memory ability than RNN, it can store long-term memories of the training data, and LSTM can approach the optimal value more stably.

In this paper, the filling method of LSTM neural network is to make up a window with 200 original values to fill the unknown value for the next step. The window move forward one step the filling value was added at the end, and delete the original value at the beginning to form a new window with 200 values. Continuously push the window forward in this way, until all the fillings you get. Since there are 144 pieces of data in just one day, single-step predictions is of little significance in this paper. The filling results in the article are all multi-step predictions.

**Figure 7:**
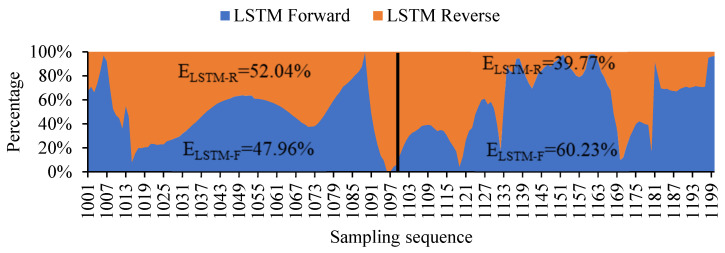
Percentage stacked area chart.

However, with the decrease of the original value and the increase of the filling value, such a filling method will lead to the accumulation of errors, making the filling error gradually increase with the translation of the window and the trend of the filling value deviates from the original value. The filling error of the LSTM one-way model is calculated, and its error distribution is shown in Figure 7. It can be clearly seen from the figure that the filling error of the forward model in the sequence of 1001 to 1100 is slightly smaller than that of the reverse model, and the filling error of the reverse model in the sequence of 1200 to 1101 is significantly smaller than that of the forward model. Based on the distribution law of the filling error of the LSTM one-way model, the two-way LSTM model can be considered.

NLP (Natural Language Processing) is an important research direction in the field of computer science and artificial intelligence. In this paper, the idea of using LSTM to fill plant stem water data was inspired by NLP training. In the field of NLP, LSTM makes up for the shortcoming that RNN cannot recognize long-distance sentences. But NLP training is different from the situation in this article: the amount of data required in NLP training is much larger than the existing data in this article, and the language information is more complicated than the stem water time series data. To a large extent, this article needs a method that is as light and fast as possible while solving the accumulation of errors. Inspired by bidirectional semantic recognition, this paper innovatively adopts bidirectional filling. Two groups of filling results are weighted to generate new and more accurate filling values to make up for the cumulative errors in the filling process.

According to the different weights of the LSTM unidirectional model, two bidirectional LSTM models were proposed in this experiment, namely the LSTM bidirectional equal weight model and the LSTM bidirectional decreasing weight model. Let the sequence number of the missing sample be *i*, the predictive value of the LSTM forward model is *x_i_*, the predictive value of the LSTM reverse model is *y_i_*, the predictive value of the LSTM bidirectional model is *z_i_*, and *a_i_*, *b_i_* are the weights of *x_i_* and *y_i_*, respectively. Therefore, for the LSTM two-way equal-weight model:

(6) $$z_{i}\;=0.5x_{i}+0.5y_{i}$$

where *i*∈(1001:1:1200).

For the LSTM bidirectional decreasing weight model:

(7) $$z_{i}\;=a_{i}x_{i}+b_{i}y_{i}$$

where *i*∈(1001:1:1200), *a_i_*∈(1:0.05:0.05), *b_i_*∈(0:0.05:0.95), 0.05 is the gradient value.

**Figure 8:**
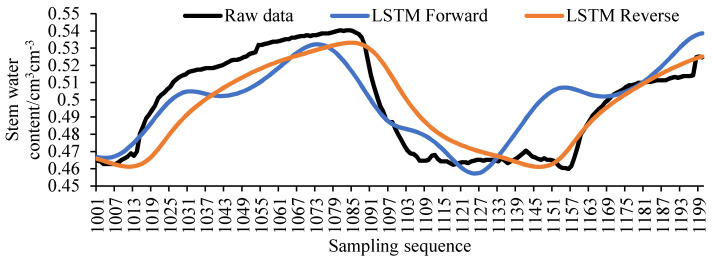
The LSTM unidirectional model.

**Figure 9:**
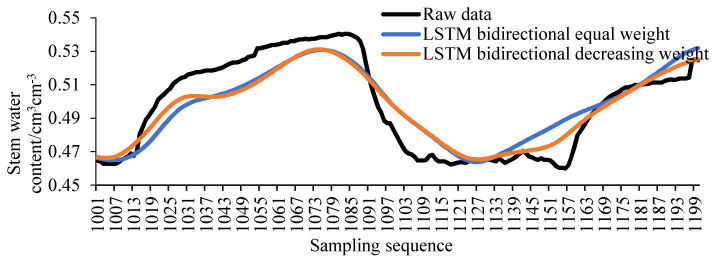
The LSTM bidirectional model.

The filling result of the unidirectional LSTM model is shown in Figure 8. The filling result of the bidirectional LSTM model is shown in Figure 9. Comparing Figure 8 and Figure 9, it can be found that the filling result of the bidirectional LSTM model is superior to the unidirectional LSTM model in the entire sequence. And the filling result of LSTM bidirectional decreasing weight model is also better than LSTM bidirectional equal weight model in the whole sequence.

**Figure 10:**
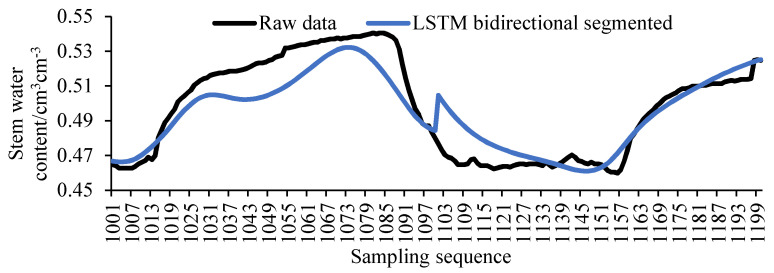
The LSTM bidirectional segmented model.

Based on the unidirectional LSTM model, this experiment also proposed a LSTM bidirectional segmented model. That is, the first half of the missing sequence (1001 to 1100) is filled with the LSTM forward model, and the second half of the missing sequence (1101 to 1200) is filled with the LSTM reverse model. The filling result of the LSTM bidirectional segmented model is shown in Figure 10. It can be seen from the figure that the fill value of the LSTM bidirectional segmented model has a large fluctuation at the intersection, which is not conducive to later data analysis.

In order to quantitatively evaluate the filling performance of the above five different LSTM models, based on the original values, the five model error parameters were calculated, and the results are shown in Table 4. It can be clearly seen from the table that the filling value of the LSTM bidirectional decreasing weight model has the smallest error. In the calibration test of the plant stem moisture sensor, the average measurement error of the sensor is 0.008 cm^3^ cm^−3^, and the average error of the LSTM bidirectional decreasing weight model is 0.009 cm^3^ cm^−3^, the two are very close, and the filling accuracy can basically meet the subsequent tests analysis. Therefore, the LSTM bidirectional decreasing weight model can be used to fill the missing sampling sequence.

**Figure 11:**
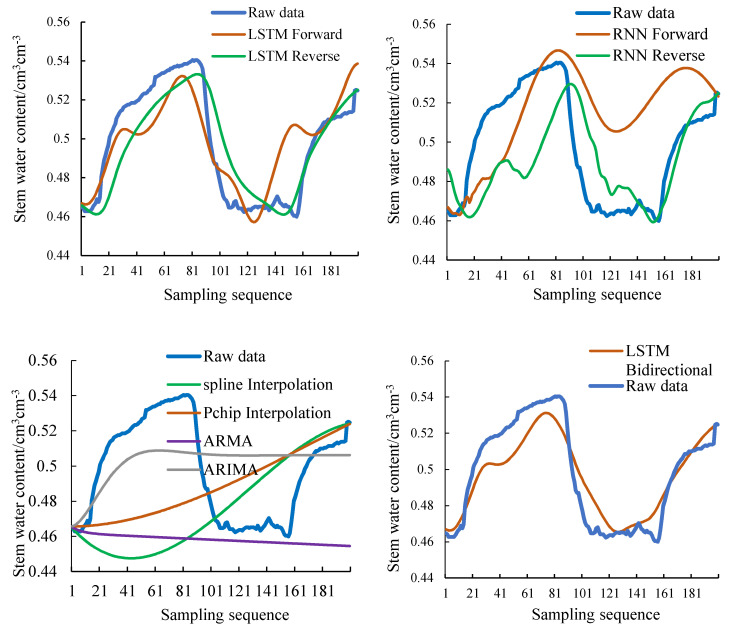
Filling results for different methods (LSTM, RNN, interpolation, LSTM bidirectional).

Figure 11 compares the filling results of several models with the original data. As can be seen from Figure 11, the fitting degree between the filling value of LSTM and the original data is better than that of RNN, interpolation, ARMA, and ARIMA. The specific error values of each model are shown in Table 5.

Table 5 shows the error parameters of several models. In order to remove the influence of the variable amplitude on the error parameters, the error calculation results are as follows:

(8) $$e=\frac{\left|x_{p}-x\right|}{x},$$

*e* is the error value. $x_{p}$ is the filling result. *x* is the true value. In Table 5, the error values of the bidirectional LSTM model are the smallest among several models, and the MAPE is 1.813%, less than the filling error of the one-way LSTM model, 1/3 of the filling error of the RNN model, and 1/4 of the interpolation filling error. The performance of ARIMA is better than that of ARMA, but the error is still larger than that of the LSTM model.

In order to show the error distribution, the number of filling errors less than the fixed value of each model was counted, and the results were shown in Table 6.

Table 6 shows the proportion of the filling error of each model less than the set value, and the conditions are not more than 0.01, 0.02, 0.03, 0.04, and 0.05, respectively. It can be seen from Table 6 that the accuracy of LSTM unidirectional and LSTM bidirectional filling results is much better than other models, and the bidirectional filling accuracy after adding weight processing is the highest. Of the points, 100% in the bidirectional filling result satisfy the error within 5%. Even if the required error is within 2%, more than half of the points still meet the requirements. The LSTM unidirectional filling performance is also excellent, but the small part of the point deviation is large. LSTM unidirectional filling is 9.5% less than the number of bidirectional LSTM in less than 5%, but still more than 90%. The error of RNN and interpolation method is very large, and accuracy 60% cannot be achieved under the requirement of error less than 5%, which makes it difficult to meet the requirement of data filling. The performance of ARIMA is the best in the traditional method, and the total error is less than 5%.

The models and parameters involved in Section 3.1 are summarized in Table 7.

Combining the data in Table 6 with Table 5, for the data of the longer sequence to be filled, the traditional data filling method has difficulty meeting the demand, and neither the degree of fitting nor the specific error-index can be compared with the filling method added to the neural network. The advantage of LSTM is more obvious than that of RNN. The ARIMA method is the best among several traditional methods, but it cannot fit data fluctuations well. The accuracy performance is not as good as LSTM.

#### 3.1.2. Model Results and Analysis after Increasing the Training Data

**Figure 12:**
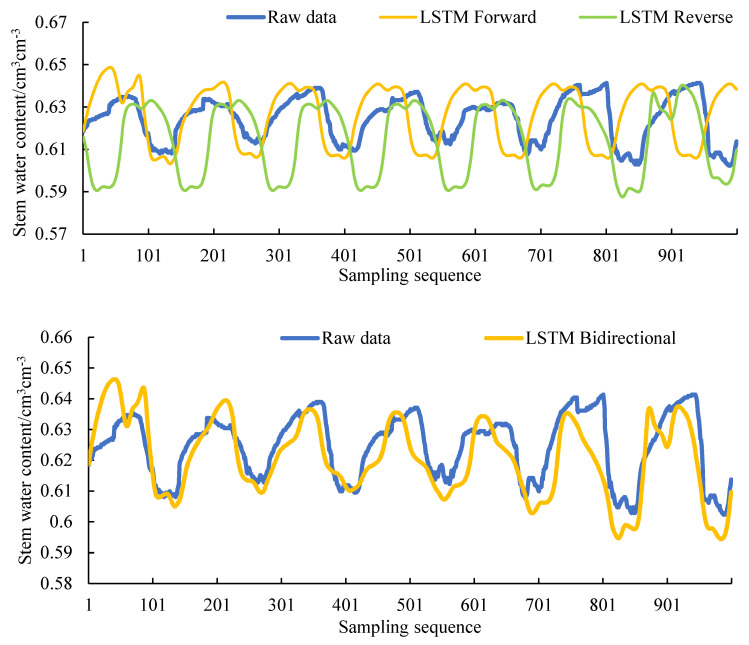
The 5000 training data filling results compared with the original data.

The training data in Section 3.1.1 is small, only 1000 pieces. In order to verify the influence of the length of the training data on the training results, 11,000 pieces of data were selected in the same database for experiments. Divided 11,000 data into the same forward and reverse as in Section 3.1.1. Using 5000 forward data and 5000 reverse data to fill in the middle 1000 missing data. Maintain a 5:1 ratio of training data to test data. The 5000 pieces of data exceeded the data length of 1 month, and 1000 pieces of data are approximately 1 week of data length. Such a data scale can better support the research results. The filling result is shown in Figure 12.

By comparing Table 5 and Table 8, it can be seen that after the training set is enlarged, the filling accuracy has been significantly improved. The sensor measurement error of 0.8% makes the filling error too small to be of no practical significance at this stage. But the results further proved the applicability of the model for stem moisture data filling, and the accuracy fully met the expected indicators. When the stem moisture value is known to be continuous and complete, the accuracy of the one-dimensional data filling model can fully meet the demand.

But when the stem moisture is intermittent and the data length is not enough to complete the filling, it is necessary to infer the stem moisture data through the introduction of environmental parameters. The accuracy of the LSTM bidirectional filling method is better than that of the one-way filling method, indicating that the error accumulation problem still exists. The accuracy will decrease as the filling sequence increases. To deal with the situation that the missing data is too long, the one-dimensional filling method still has its limitations.

### 3.2. Multidimensional LSTM Model Results Analysis

In order to solve the problems mentioned in the previous section, this section tries a multi-dimensional stem moisture filling method. Multi-dimensional data filling is based on multi-dimensional data correlation and is not affected by sequence length. Derive the moisture value of the stem according to the law of environmental parameters. Especially when the stem moisture data is intermittently incomplete and a long complete sequence cannot be obtained, the advantage is especially obvious.

**Figure 13:**
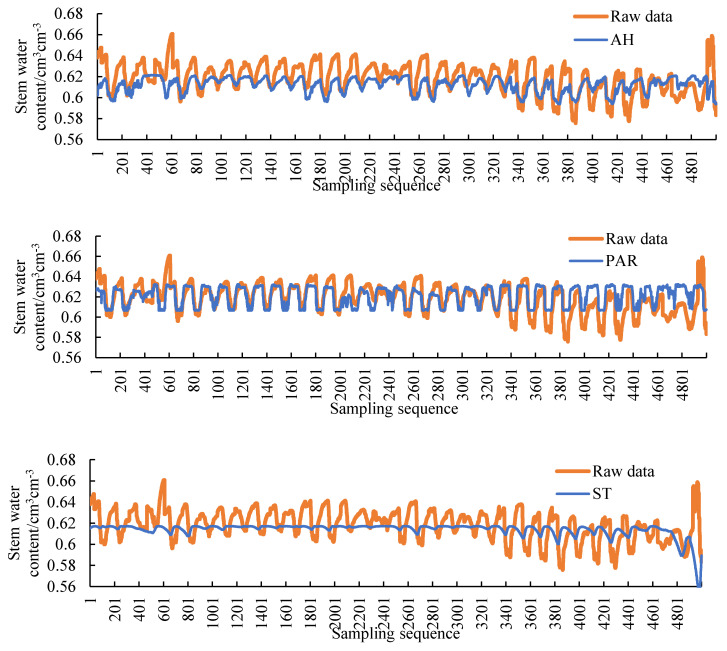

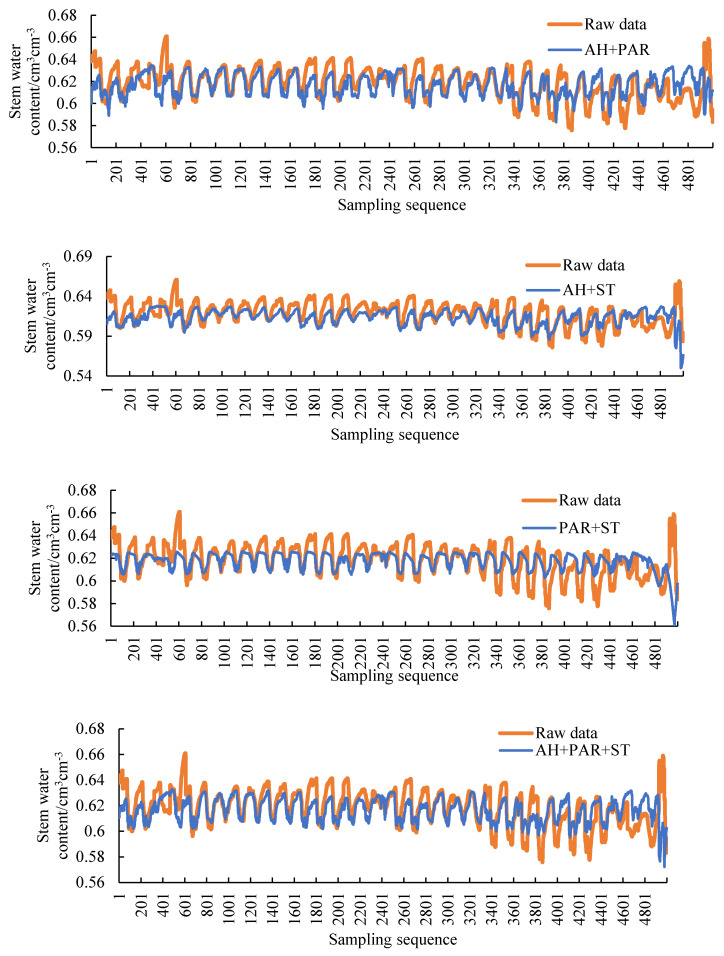
Filling result curves of different inputs (air humidity (AH), photosynthetically active radiation (PAR), soil temperature (ST), AH + PAR, AH + ST, PAR + ST, AH + PAR + ST).

Three environmental parameters, soil temperature, air humidity, and photosynthetically active radiation, which are highly correlated with plant stem moisture, are selected as input values, and stem moisture is used as an output value. The correlations are shown in Table 9. In order to select the model structure with the best filling result, the air humidity (AH), photosynthetically active radiation (PAR), soil temperature (ST), AH + PAR, AH + ST, PAR + ST, AH + PAR + ST are selected separately. Ensured that other conditions are the same. The filling result is shown in Figure 13 and the error parameters are shown in Table 10.

By reading Figure 13 and Table 10, the smallest errors in the filling results are PAR and PAR + ST and AH + PAR + ST. The errors of the three types are similar, and the MAE value is less than 1%, which meets the requirements of filling accuracy. The error parameter of PAR is the smallest, but it can be seen from the figure that the prediction result does not fit the fluctuation trend well. The error parameter of AH + PAR + ST is larger than PAR, but it can better fit the fluctuation trend. In the process of stem moisture research, the changing trend is very important. When the three models meet the accuracy requirements, the AH + PAR + ST model with better trend fitting is selected for subsequent filling.

**Figure 14:**
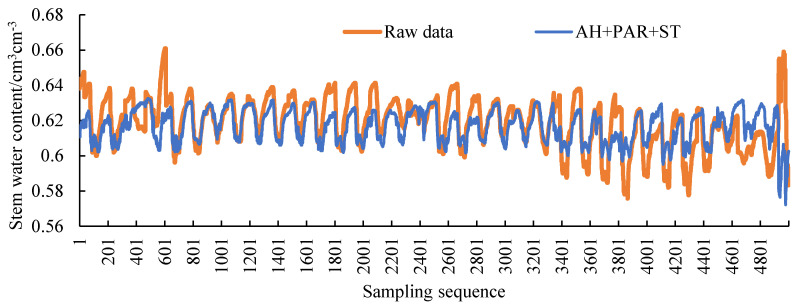
The comparison between the filling result of the third model with the smallest error value and the actual value.

The 5000 sets of stem moisture and corresponding environmental parameter data of the same plant in the one-dimensional model were selected as the training set, and three different models were constructed: The 2-layer LSTM network has 50 neurons per layer; The 3-layer LSTM network has 50 neurons per layer; The 3-layer LSTM network has 50 neurons per layer and the dropout weight is set to 0.8. The stem moisture values of length 5000 were filled by using three models, and the filling results were compared with the known true values. The error results are shown in Table 11. The statistical results of the three models with errors not exceeding 0.01, 0.02, 0.03, 0.04, and 0.05 are listed in Table 12. The comparison between the filling result of the third model with the smallest error value and the actual value is shown in Figure 14.

Table 11 shows that the error minimum model is a three-layer LSTM model with 0.8 dropout weights. The accuracy of the filling result is similar to that of the LSTM bidirectional filling model in Table 5. The error distribution of the multi-dimensional filling model in Table 12 shows that 90% of the error is less than 0.03, 44% is less than 0.01, and the number of large errors is small. While the accuracy meets the demand, the filling value curve is closely fitted to the true value curve.

The length of the filling sequence of the one-dimensional filling model is limited. The longer the sequence, the greater the accumulation of errors. The multi-dimensional filling method is to fill the stem moisture data according to environmental parameters. If the environmental parameters are complete, the length of the data to be filled is not limited. And to a certain extent, it can cope with the violent fluctuation of stem moisture caused by environmental changes.

## 4. Conclusions

High-precision data filling can deal with data missing due to various reasons. Data integrity is the basis of data analysis. Stem moisture is data that has periodic and non-periodic characteristics affected by various environmental factors. In order to better cope with the long-term data loss caused by the failure of sensors, this paper proposes a method of filling missing data based on LSTM. By comparing the traditional interpolation method, time series statistical model, one-dimensional LSTM method, and the LSTM method with environmental parameters, the results show that the one-dimensional LSTM filling model has the highest accuracy, and the LSTM method with environmental parameters has a longer filling sequence and better versatility. The experiment in this paper verifies the feasibility of LSTM model in data filling, and the introduction of environmental parameters improves the filling of stem moisture data. Deep learning methods combined with appropriate data processing can improve prediction accuracy.

Specific examples show the following:

(1) In the long-term data loss field, the traditional data filling method cannot cope with the fluctuation phenomenon in the missing data. Only using the time series statistical model, the fitting accuracy of the filling results cannot meet the requirements. The neural network filling model has great advantages in data filling compared with the traditional method, and it is helpful for filling the data missing for a long time.
(2) In this paper, the bidirectional filling method based on the LSTM model has an obvious effect on reducing the influence of cumulative errors in long-term filling and further improves the accuracy of data. The MAPE was 1.813%, and the MAE was 0.909%. However, the error accumulation problem of one-dimensional prediction cannot be avoided, and the length of the filling sequence is limited.
(3) Increasing the amount of training data can improve the accuracy of the filling results. Increase the amount of training data to 5000 and the accuracy is significantly improved. The MAE value is 0.626%, and the MAPE value is 1.001%. Although there is still a problem of error accumulation, the short-term filling accuracy has fully met the requirements.
(4) In order to further expand the data length and solve the error accumulation problem, the multidimensional data filling model is built using environmental parameters. The filling results prove that data filling using the model is not affected by the sequence length, and there is no requirement for the location of the missing data. The MAPE was 1.499%, and the MAE was 0.930%. It enhances the versatility of the model while ensuring accuracy.

In the future, we will try to combine time series statistical models with deep learning methods, such as combining ARIMA with LSTM and GRU models to improve accuracy while enhancing the interpretability of the model. Fully consider the periodic characteristics of the data. Analyze cyclical and non-cyclical components to take advantage of different models. The accuracy can be further improved [31]. Considering the influence of the data interval, choosing an appropriate data interval is beneficial to improve the accuracy of the results [27].

## Figures and Tables

**Table 1:** LSTM Network Model Overview.

Layer (Type)	Output Shape	Parameter
lstm_1 (LSTM)	(100, 200, 256)	264,192
dropout_1(Dropout)	(100, 200, 256)	0
lstm_2 (LSTM)	(100, 200, 128)	197,120
lstm_3 (LSTM)	(100, 64)	49,408
dense_1 (Dense)	(100, 1)	65

**Table 2:** LSTM Network Model Overview.

Layer (Type)	Output Shape	Parameter
simple_rnn_1 (SimpleRNN)	(100, 200, 256)	66,048
dropout_1(Droput)	(100, 200, 256)	0
simple_rnn_2 (SimpleRNN)	(100, 200, 128)	49,280
simple_rnn_3 (SimpleRNN)	(100, 64)	12,352
dense_1 (Dense)	(100, 1)	65

**Table 3:** Data source information.

Species	Sampling Period	Location	Sensor	Length
*Lagerstroemia Indica*	10 mins	Haidian district of Beijing	BD-IV plant stem moisture sensor	2200

**Table 4:** The prediction error of LSTM models.

Type	Maximum/%	Minimum/%	MAE/%	MAPE/%	RMSE/%
Forward	10.229	0.014	1.274	2.571	1.637
Reverse	6.169	0.005	1.072	2.153	1.373
Bidirectional equal weight	6.360	0.021	1.067	2.139	1.267
Bidirectional decreasing weight	4.557	0.005	0.909	1.813	1.094
Bidirectional segmented	6.155	0.014	0.951	1.893	1.206

**Table 5:** Comparison of error statistical results.

Type	Direction	Maximum/%	Minimum/%	MAE/%	MAPE/%	RMSE/%
LSTM	Forward	4.704	0.007	1.274	2.571	1.637
	Reverse	3.013	0.003	1.072	2.153	1.373
RNN	Forward	6.730	0.027	2.912	6.011	3.440
	Reverse	5.146	0.010	2.055	4.063	2.599
Spline interpolation		8.501	0.007	3.845	7.518	4.857
Pchip interpolation		6.185	0.024	3.142	6.217	3.746
ARMA		8.165	0.028	3.940	7.630	4.816
ARIMA		4.616	0.000	2.307	4.709	2.697
LSTM bidirectional		2.117	0.002	0.909	1.813	1.094

**Table 6:** Comparison of error distributions.

Type	Direction	Less Than 1%/%	Less Than 2%/%	Less Than 3%/%	Less Than 4%/%	Less Than 5%/%
LSTM	Forward	24.5	50	66	86	90.5
	Reverse	31.5	56	76	81.5	86.5
RNN	Forward	14.5	23.5	28	33.5	40.5
	Reverse	18	32	48.5	54	60
Spline interpolation		7.5	24.5	31	37	44
Pchip interpolation		10	25.5	29	33	38
ARMA		29.5	37	42	47	54
ARIMA		26	50	64.5	80	100
LSTM bidirectional		34.5	56.5	78	95.5	100

**Table 7:** Summary table of all model parameters.

Type	Direction	Input	Output	Number of Neurons in Each Layer	Training Algorithm	Epoch	Batch Size
LSTM	Forward	1000	200	3layer:256*128*64	LSTM	100	100
	Reverse	1000	200	3layer:256*128*64	LSTM	100	100
RNN	Forward	1000	200	3layer:256*128*64	RNN	100	100
	Reverse	1000	200	3layer:256*128*64	RNN	100	100
LSTM Bidirectional		1000/1000 ^1^	200/200 ^2^	3layer:256*128*64	LSTM	100	100

^1,2^ Bidirectional filling has two sets of input and output, which are forward and reverse, respectively.

**Table 8:** Comparison of error statistical results.

Type	Direction	Maximum/%	Minimum/%	MAE/%	MAPE/%	RMSE/%
LSTM	Forward	2.160	0.004	0.737	1.179	0.938
LSTM	Reverse	3.946	0.005	2.217	3.547	2.544
LSTM	Bidirectional	1.952	0.001	0.626	1.001	0.847

**Table 9:** Correlations between poplar stem water content and micro-environment parameter set.

Type	Soil Temperature	Soil Moisture	Air Temperature	Air Humidity	Photosynthetically Active Radiation
Stem moisture 1	0.278	0.188	0.009	0.564	−0.291
Stem moisture 2	0.284	0.211	−0.001	0.579	−0.308
Stem moisture 3	0.302	0.241	0.011	0.572	−0.322
Stem moisture 4	0.306	0.262	0.031	0.564	−0.304

**Table 10:** Error list for different inputs.

Type	Maximum/%	Minimum/%	MAE%	MAPE/%	RMSE/%
AH	5.678	0	1.133	1.817	1.403
PAR	3.947	0	0.895	1.455	1.171
ST	9.913	0.002	1.258	2.025	1.635
AH + PAR	6.458	0	1.015	1.642	1.329
AH + ST	7.892	0	1.062	1.703	1.407
PAR + ST	9.522	0.001	0.918	1.482	1.315
AH + PAR + ST	7.69	0	0.93	1.499	1.277

**Table 11:** Comparison of error statistical results.

Type	Maximum/%	Minimum/%	MAE%	MAPE/%	RMSE/%
2*50	12.187	0.001	1.18	1.904	1.797
3*50	8.235	0	1.0314	1.67	1.315
3*50*0.8	7.69	0	0.93	1.499	1.277

**Table 12:** Comparison of error distributions.

Type	Less Than 1%/%	Less Than 2%/%	Less Than 3%/%	Less Than 4%/%	Less Than 5%/%
2*50	39.72	66.28	81.48	90.18	94.38
3*50	41.12	69.22	85.92	93.54	95.88
3*50*0.8	44.12	71.10	90.90	95.46	97.74
